# TNF‐α has both stimulatory and inhibitory effects on mouse monocyte‐derived osteoclastogenesis

**DOI:** 10.1002/jcp.26024

**Published:** 2017-07-17

**Authors:** Yixuan Cao, Ineke D.C. Jansen, Sara Sprangers, Teun J. de Vries, Vincent Everts

**Affiliations:** ^1^ Department of Oral Cell Biology and Functional Anatomy Academic Center for Dentistry Amsterdam (ACTA) University of Amsterdam and Vrije Universiteit Amsterdam Amsterdam The Netherlands; ^2^ Department of Periodontology Academic Center for Dentistry Amsterdam (ACTA) University of Amsterdam and Vrije Universiteit Amsterdam Amsterdam The Netherlands

**Keywords:** monocyte, osteoclast, tumor necrosis factor α

## Abstract

Phenotypically different osteoclasts may be generated from different subsets of precursors. To what extent the formation of these osteoclasts is influenced or mediated by the inflammatory cytokine TNF‐α, is unknown and was investigated in this study. The osteoclast precursors early blasts (CD31^hi^Ly‐6C^−^), myeloid blasts (CD31^+^Ly‐6C^+^), and monocytes (CD31^−^Ly‐6C^hi^) were sorted from mouse bone marrow using flow cytometry and cultured with M‐CSF and RANKL, with or without TNF‐α. Surprisingly, TNF‐α prevented the differentiation of TRAcP^+^ osteoclasts generated from monocytes on plastic; an effect not seen with early blasts and myeloid blasts. This inhibitory effect could not be prevented by other cytokines such as IL‐1β or IL‐6. When monocytes were pre‐cultured with M‐CSF and RANKL followed by exposure to TNF‐α, a stimulatory effect was found. TNF‐α also stimulated monocytes’ osteoclastogenesis when the cells were seeded on bone. Gene expression analysis showed that when TNF‐α was added to monocytes cultured on plastic, RANK, NFATc1, and TRAcP were significantly down‐regulated while TNF‐αR1 and TNF‐αR2 were up‐regulated. FACS analysis showed a decreased uptake of fluorescently labeled RANKL in monocyte cultures in the presence of TNF‐α, indicating an altered ratio of bound‐RANK/unbound‐RANK. Our findings suggest a diverse role of TNF‐α on monocytes’ osteoclastogenesis: it affects the RANK‐signaling pathway therefore inhibits osteoclastogenesis when added at the onset of monocyte culturing. This can be prevented when monocytes were pre‐cultured with M‐CSF and RANKL, which ensures the binding of RANKL to RANK. This could be a mechanism to prevent unfavorable monocyte‐derived osteoclast formation away from the bone.

## 1 INTRODUCTION

An increasing number of people throughout the world suffer from inflammation‐related bone diseases, such as rheumatoid arthritis and periodontitis. Excessive release of inflammatory cytokines is associated with chronic inflammation and bone destruction. Tumor necrosis factor α (TNF‐α) is one of the most prominent inflammatory cytokines, and therefore, has been targeted in therapies against bone diseases (Bingham, 2002).

Osteoclasts, the multinucleated bone‐resorbing cells, are crucial in bone diseases with excessive bone loss. Severe bone destruction occurs when the equilibrium of osteoclast and osteoblast activity is disturbed. Osteoclasts arise from monocytic precursors under the influence of M‐CSF and RANKL. Next to these cytokines, TNF‐α has been shown to stimulate osteoclast generation and bone resorption both in vitro (Thomson, Mundy, & Chambers, 1987) and in vivo (König, Mühlbauer, & Fleisch, 1988). TNF‐α‐ and RANKL‐induced osteoclastogenesis share a similar intracellular pathway (Kitaura et al., 2013). They both induce osteoclast differentiation by activating c‐fos and NFATc1 signaling (Yamashita et al., 2007). TNF‐α induces TRAF2, which can further stimulate RANK associated TRAF6‐induced osteoclastogenesis (Kitaura et al., 2013).

TNF‐α recognizes two receptors both in human and in mouse, TNF‐R1 (p55) and TNF‐R2 (p75) (Vandenabeele, Declercq, Beyaert, & Fiers, 1995). TNF‐R1 promotes osteoclastogenesis (Abu‐Amer et al., 2000) and the stimulatory effect of TNF‐α can be completely prevented by anti‐p55 antibody. Blocking of TNF‐R2 with anti‐p75 antibody only partially inhibits osteoclastogenesis (Azuma, Kaji, Katogi, Takeshita, & Kudo, 2000; Kobayashi et al., 2000). TNF‐α accelerates RANKL‐induced osteoclastogenesis via coupling to TNF‐α R1 (Zhang, Heulsmann, Tondravi, Mukherjee, & Abu‐Amer, 2001). Although some studies showed that TNF‐α is RANK/RANKL‐dependent (Lam et al., 2000), others showed that TNF‐α induced osteoclastogenesis is independent of RANK/RANKL (Kim et al., 2005; Kobayashi et al., 2000).

Cells that can differentiate into osteoclasts are widely distributed in the body, including bone marrow precursors, peritoneal macrophages, splenocytes, peripheral blood‐borne monocytes, and dendritic cells (Marks & Walker, 1981; Quinn, Sabokbar, & Athanasou, 1996; Rivollier et al., 2004; Scheven, Visser, & Nijweide, 1986). Several studies have shown that osteoclast precursors isolated from different skeletal sites are not always identical in terms of osteoclastogenesis (Azari, Schoenmaker, de Souza Faloni, Everts, & De Vries, 2011; De Souza Faloni et al., 2011; Everts, de Vries, & Helfrich, 2009). Even within the same site, different precursor subsets were shown to differ in their capacity to form osteoclasts (Cao et al., 2016; De Vries, Schoenmaker, Hooibrink, Leenen, & Everts, 2009; De Vries et al., 2015; Jacquin, Gran, Lee, Lorenzo, & Aguila, 2006; Sprangers, Schoenmaker, Cao, Everts, & de Vries, 2016).

One of the main sites where osteoclast precursors reside is the bone marrow. In the marrow of mice three monocytic precursors can be recognized: early blasts (CD31^hi^ Ly‐6C^−^), myeloid blasts (CD31^+^ Ly‐6C^+^), and monocytes (CD31^−^ Ly‐6C^hi^) (Nikolic, de Bruijn, Lutz, & Leenen, 2003). Each subset has the potential to differentiate into osteoclasts (De Vries et al., 2009). Recently, studies have reported that these three precursor subsets respond differently to the growth factor M‐CSF (De Vries et al., 2015) and the cytokine IL‐1β (Cao et al., 2016). Myeloid blasts were found to respond the fastest to M‐CSF and RANKL (De Vries et al., 2009) and early blasts was the only population that proliferated under the influence of IL‐1β (Cao et al., 2016). How these three myeloid lineage‐osteoclast precursor subsets respond to another crucial inflammatory cytokine, TNF‐α, is unknown and is the topic of the present study. We investigated the effect of TNF‐α on the osteoclastogenic potential of different mouse bone marrow‐derived osteoclast precursors.

## 2 METHODS AND MATERIALS

### 2.1 Bone marrow cell isolation

Bone marrow cells were isolated from 6‐week‐old male C57BL/6J mice (Vrije Universiteit Amsterdam animal facility, Amsterdam, the Netherlands). Femur and tibia from both legs were removed, followed by grinding in a mortar and cells were suspended in 10 ml culture medium. Culture medium was α‐MEM (Gibco; Thermo Fisher Scientific, Paisley, Scotland) supplemented with 5% Fetal Clone I Serum (HyClone; GE Healthcare Life Sciences, Logan, UT) and 1% penicillin‐streptomycin‐fungizone (Sigma–Aldrich, St. Louis, MO). The cell suspension was aspirated through a 21 gauge needle and filtered through 40 µm filter. A total of 4 × 10^7^ cells per mouse were collected. Animal experiments were approved by the Animal Welfare Committee of the Vrije Universiteit Amsterdam.

### 2.2 Immunofluorescence labeling and cell sorting

Bone marrow cells were labeled with CD31 and Ly‐6C following the labeling procedure described previously (Cao et al., 2016). In brief, cells were first labeled with biotinylated CD31 antibody (AbD Serotec, Kidlington, United Kingdom) for 30 min, and subsequently with Alexa 488‐conjugated Ly‐6C (AbD Serotec) and streptavidin‐PE (Becton Dickinson, San Jose, CA) for 30 min. Cells were washed in FACS buffer (PBS containing 1% BSA) before sorting. Early blasts (CD31^hi^ Ly‐6C^−^), myeloid blasts (CD31^+^ Ly‐6C^+^), and monocytes (CD31^−^ Ly‐6C^hi^) were sorted with FACSAria (Becton Dickinson).

### 2.3 Cell culturing

The three sorted subsets, early blasts, myeloid blasts, and monocytes were seeded in 96‐well plastic plates (CELLSTAR®; Greiner Bio‐One, Monroe, NC) at a density of 1.5 × 10^4^ cells/well in 150 μl culture medium with different conditions: with or without 30 ng/ml M‐CSF (R&D Systems, Minneapolis, MN), with or without 20 ng/ml RANKL (RANKL‐TEC, R&D systems), with 0, 10, or 100 ng/ml TNF‐α (R&D Systems), with or without 10 ng/ml IL‐1β (Sigma–Aldrich), with or without 10 ng/ml IL‐6 (Sigma–Aldrich). Cells were cultured on plastic or on 650 μm‐thick bone slices and culture media were refreshed every 3 days. After 5 days the cultures on plastic plates were stopped by fixation in 4% PBS‐buffered formaldehyde for TRAcP analysis, or lysed in RNA lysis buffer (Qiagen, Hilden, Germany) at day 0, 3, and 5 for RNA isolation. Bone slices were stored in water after 6 days of culture and culture media was collected for CTX assay.

### 2.4 Tartrate‐resistant acid phosphatase (TRAcP) staining

Osteoclasts were identified as TRAcP^+^ multinucleated cells by using a commercially available Leucocyte acid phosphatase kit (Sigma–Aldrich). The staining procedure was performed following the manufacturer's instructions. Nuclei were counterstained by 4′6‐diamidino‐2‐phenylindole (DAPI). The TRAcP^+^ multinucleated cells were categorized as cells containing 3–10, and >10 nuclei, and the number of cells in each category was counted using a combination of light and fluorescence microscopy (Leica DFC320; Leica Microsystems, Wetzlar, Germany).

### 2.5 Bone resorption

In order to evaluate bone resorption, formation of resorption pits on bone slices was analyzed by Coomassie brilliant blue staining. After 6 days of culture, bone slices were washed with water and the cells were removed by sonication for 30 min in 10% ammonia (Merck, Darmstadt, Germany) on ice. To reduce staining background the bone surface was incubated in saturated alum for 10 min and subsequently washed with water. Resorption pits were stained with Coomassie brilliant blue (Pharmacia, Uppsala, Sweden) and visualized by light microscopy (Leica DFC320). In addition, CTX concentrations of the culture supernatants were determined using CrossLaps® for culture ELISA (Immunodiagnostic Systems Limited, Frankfurt am Main, Germany). Culture supernatants were collected after 6 days and CTX assay was conducted following the manufacturer's instructions.

### 2.6 Quantitative RT‐PCR

The procedure of RT‐PCR was described in detail in a previous paper (Cao et al., 2016). Quantitative expression of TRAcP, NFATc1, RANK, TNF‐αR1, and TNF‐αR2 were determined. Primers were designed using Primer Express 3.0.1: TRAcP, Fw: gACAAgAggTTCCAggAgACC, Rv: gggCTggggAAgTTCCAg; NFATc1, Fw: CATgCgAgCCATCATCgA, Rv: TgggATgTgAACTCggAAgAC; RANK, Fw: TgggCTTCTTCTCAgATgTCTTT, Rv: TgCAgTTggTCCAAggTTTg; TNF‐αR1, Fw: TTgCAgCCACTgCAAgAAAA, Rv: ACAgCACCgCAgTACCTgAgT; TNF‐αR2, Fw: TgCCAgATCTCACAggAATACTATg, Rv: TCCgAggTCTTgTTgCAgAA. PBGD (housekeeping gene), Fw: AgTgATgAAAgATgggCAACT, Rv: TCTggACCATCTTCTTgCTgA. The relative expression of each gene was calculated as 2^−ΔCt^, ΔCt = C _gene of interest _ −Ct _PBGD_. The results were shown as fold increased, normalized by the same gene expression at day 0.

### 2.7 FACS analysis

After the isolation of myeloid blasts and monocytes, these precursor cells were cultured with M‐CSF and RANKL, with or without 10 ng/ml TNF‐α for 3 days. Subsequently, precursor cells were collected using cell dissociation solution (Sigma–Aldrich). To analyze the capacity of cells to take up RANKL, cells were incubated with TAMRA conjugated‐recombinant murine‐RANKL for 30 min. TAMRA labeled (rm)RANKL stock was prepared by mixing 5 mg 5(6)‐TAMRA, SE (Molecular probes, Thermo Fisher Scientific) with 10 μg (rm)RANKL (R&D Systems) and incubated for 2 hr at room temperature. Purification was conducted by ultimate 3000 HPLC (Thermo Scientific and RPC‐8‐Vaydac column [GRACE]), and authenticity was confirmed by mass spectrometry MALDI‐TOF Microflex (Bruker Daltonik GmbH, Bremen, Germany). For RANK expression, nonspecific binding was blocked by incubation with a combination of TruStain fcXTM Antibody (Fc receptors block) (BioLegend, San Diego, CA) in PBS supplemented with 1% BSA for 10 min. Subsequently, staining was performed using 1:100 diluted PE‐conjugated anti‐mouse CD265 (RANK) antibody (BioLegend) and PE‐conjugated IgG2a, ĸ Isotype Ctrl antibody (BioLegend) in PBS supplemented with 1% BSA for 30 min on ice. Cells were subsequently fixed with 1% formaldehyde and analyzed by FACS (BD Accuri™ C6 Flow Cytometer). Data were analyzed by CFlow® Plus software (Version 1.0.202.1).

### 2.8 Statistical analysis

All data were analyzed from six mice (*n* = 6) and GraphPad Prism (version 6.00; GraphPad Software, LaJolla, CA) was used for the statistical analysis. One‐way ANOVA followed by Tukey–Kramer's multiple comparison was used for multiple comparisons, and *t*‐test was used for two comparisons. Data are displayed as mean ± SD and *p *< 0.05 was considered as significant difference.

## 3 RESULTS

### 3.1 TNF‐α prevents monocyte‐derived osteoclastogenesis on plastic; an effect that was abolished with M‐CSF and RANKL priming

**Figure 1:**
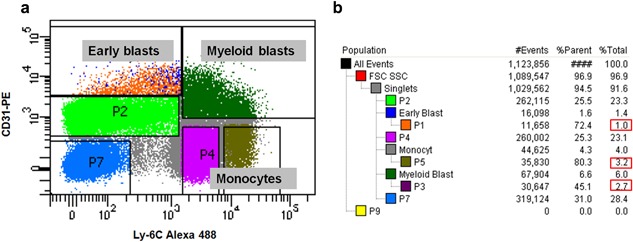
Flow cytometric gating of murine bone marrow. (a) Three osteoclast precursor subsets, early blasts, myeloid blasts, and monocytes were sorted after labeling CD31 and Ly‐6C. (b) Percentage of each population. In total six populations could be distinguished: early blasts (1.0%), myeloid blasts (6.0%), monocytes (4.0%), lymphocytes (23.3%), erythroid blasts (28.4%), and granulocytes (23.1%)

Bone marrow cells were sorted by flow cytometry and three osteoclast precursor subsets early blasts (CD31^hi^ Ly‐6C^−^), myeloid blasts (CD31^+^ Ly‐6C^+^), and monocytes (CD31^−^ Ly‐6C^hi^) were isolated according to the expression of CD31 and Ly‐6C from the whole population (Figure 1). These three osteoclast precursor subsets were cultured on plastic with M‐CSF and RANKL, without (condition 1) or with (condition 2) 10 or 100 ng/ml TNF‐α from the 1st day and cultures were analyzed after 5 days.

**Figure 2:**
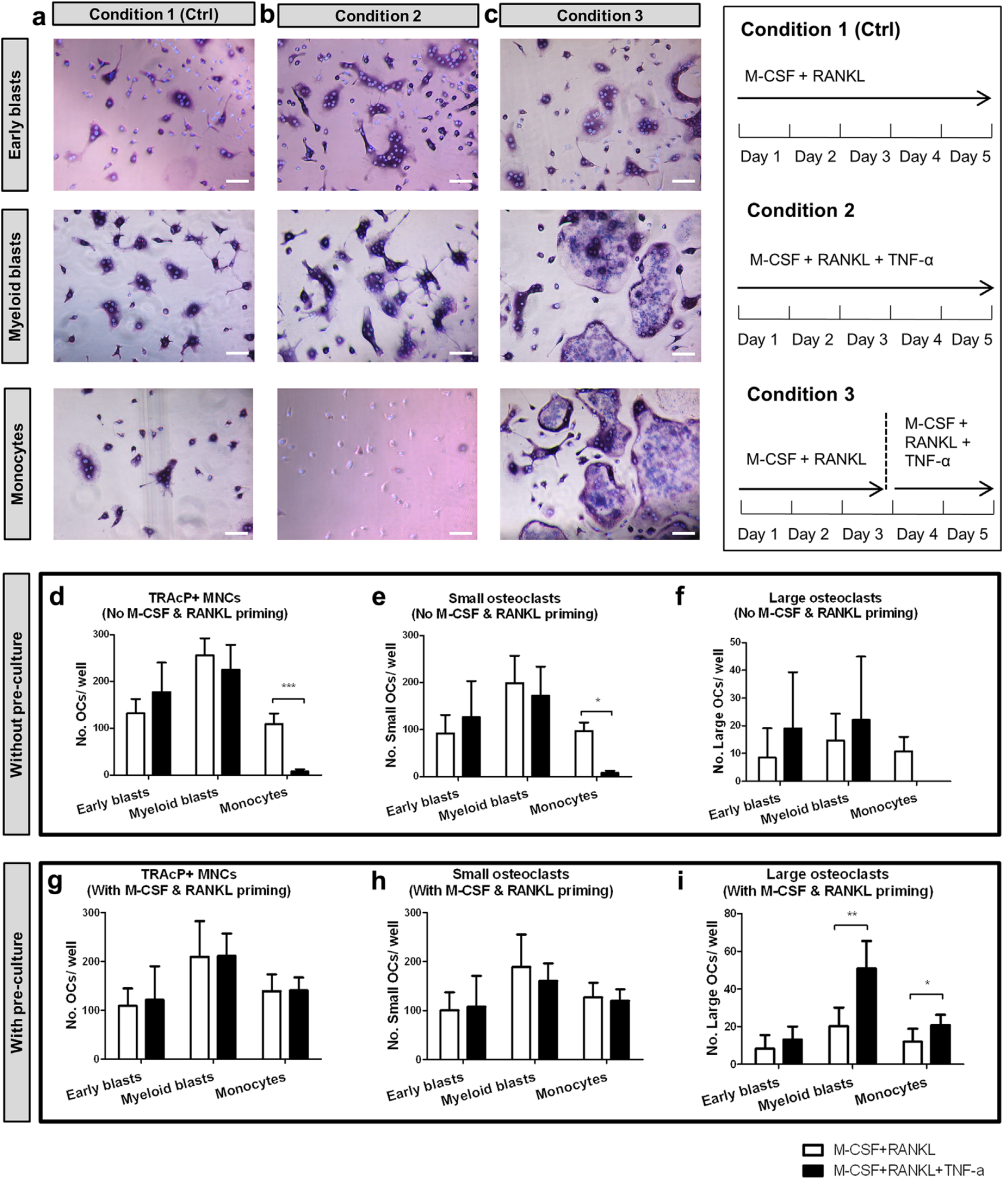
Effect of TNF‐α on osteoclastogenesis by different osteoclast precursors on plastic. (a–c) Early blasts (1st row), myeloid blasts (2nd row), and monocytes (3rd row) were cultured under three different culture conditions: (a) condition 1 (control), with 30 ng/ml M‐CSF and 20 ng/ml RANKL for 5 days; (b) condition 2, with 30 ng/ml M‐CSF, 20 ng/ml RANKL and 10 ng/ml TNF‐α for 5 days; (c) condition 3, first cultured with 30 ng/ml M‐CSF and 20 ng/ml RANKL for 3 days, then cells were further cultured with 30 ng/ml M‐CSF, 20 ng/ml RANKL and 10 ng/ml TNF‐α for 2 days. Cells were stained for TRAcP activity and nuclei were counterstained by DAPI. Osteoclasts were recognized as TRAcP^+^ multinucleated cells (purple) and nuclei were visualized as blue. Note the absence of any multinucleated cells in monocyte cultures in condition 2. Scale bar = 100 μm. (d–f). Total number of TRAcP^+^ multinucleated cells (>2 nuclei), number of small osteoclasts (3–10 nuclei), and number of large osteoclasts (>10 nuclei) generated by the three subsets. White column: cells cultured with 30 ng/ml M‐CSF and 20 ng/ml RANKL for 5 days (condition 1). Black column: cells cultured with 30 ng/ml M‐CSF, 20 ng/ml RANKL, and 10 ng/ml TNF‐α for 5 days (condition 2). (g–i). Total number of TRAcP^+^ multinucleated cells (>2 nuclei), number of small osteoclasts (3–10 nuclei), and number of large osteoclasts (>10 nuclei) generated by the three subsets with M‐CSF and RANKL priming. White column: cells cultured with 30 ng/ml M‐CSF and 20 ng/ml RANKL for 5 days (condition 1). Black column: cells first cultured with 30 ng/ml M‐CSF and 20 ng/ml RANKL for 3 days, then 10 ng/ml TNF‐α was added and cells were further cultured with M‐CSF, RANKL, and TNF‐α for 2 more days (condition 3). (*n* = 6, *p < 0.05, **p < 0.01, ***p < 0.001)

In both early blast and myeloid blast cultures, TRAcP^+^ multinucleated cells were formed (Figure 2). This was apparent with or without TNF‐α and no significant differences were found in the number of TRAcP^+^ multinucleated cells between control (condition 1) and cultures in the presence of TNF‐α (condition 2) (Figure 2d–f). In contrast herewith, in monocyte cultures, TNF‐α almost completely prevented the formation of TRAcP^+^ multinucleated cells (Figure 2b in monocyte panel and Figure 2d). Most of the monocytes remained TRAcP‐negative mononuclear cells when TNF‐α was added at the beginning together with M‐CSF and RANKL (Figure 2b in monocyte panel). As a control, we also cultured whole bone marrow cells with or without TNF‐α, and such inhibitory effect was not seen (data not shown). There was no significant difference between the two concentrations (10 and 100 ng/ml) of TNF‐α (Figure S1a–c), therefore we chose 10 ng/ml TNF‐α for the subsequent experiments.

Lam et al. (2000) reported a similar TNF‐α‐induced inhibitory effect on osteoclast formation by purified murine myeloid cells and they found that the inhibitory effect could be overcome by RANKL exposure. In their study, TNF‐α induced osteoclast formation was maximal when the cells were exposed to RANKL for 2–4 days before adding TNF‐α (Lam et al., 2000). Therefore, in the present study we cultured the precursor cells with M‐CSF and RANKL for 3 days before adding TNF‐α (condition 3) to find out if this condition could alter the response to the cytokine. This proved indeed to be the case. Under this condition the inhibitory effect of TNF‐α in the monocyte cultures was totally abolished (Figure 2c in monocyte panel, Figure 2g in monocyte column). In all cultures, the cells became TRAcP^+^ multinucleated cells (Figure 2c in monocyte panel). Now TNF‐α even proved to stimulate the formation of large osteoclasts (>10 nuclei) by myeloid blasts and monocytes (Figure 2i in myeloid blast and monocyte columns).

Next, we analyzed whether priming with M‐CSF alone (without RANKL) was sufficient to have such a TNF‐α induced effect on osteoclastogenesis. The three subsets were cultured with M‐CSF for 3 days before adding TNF‐α. Under these conditions only few TRAcP^+^ multinucleated cells were generated by early blasts and myeloid blasts, and no TRAcP^+^ cells were seen in monocyte cultures (Figure S1d). This indicates that RANKL is essential for the TNF‐α induced osteoclast formation by monocytes.

### 3.2 IL‐1β and IL‐6 were not able to prevent the inhibitory effect of TNF‐α on monocytes’ osteoclastogenesis

**Figure 3:**
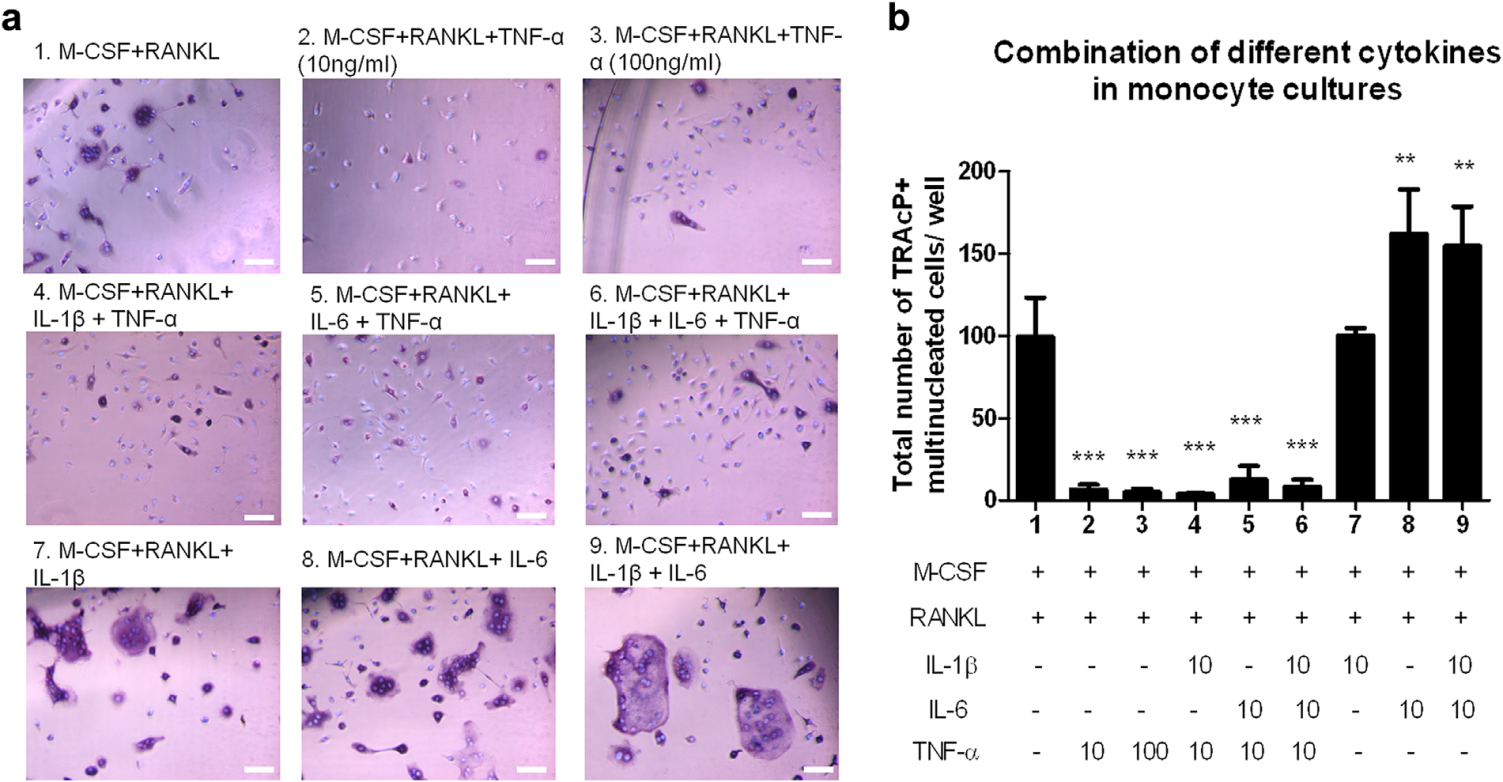
The inflammatory cytokines IL‐1β and IL‐6 cannot overcome the inhibitory effect of TNF‐α on monocytes’ osteoclastogenesis. (a) Micrographs of monocyte cultures on plastic with the combination of different cytokines. Cells were visualized with TRAcP staining (purple) and nuclei were counterstained with DAPI (blue). TRAcP^+^ cells with more than two nuclei were counted as osteoclasts. Scale bar = 100 μm. (b) Counting results of these nine different culture conditions. (*n* = 6, ** *p* < 0.01, ***p < 0.001)

Next to TNF‐α also two other inflammatory cytokines, IL‐1β and IL‐6, have been shown to stimulate osteoclastogenesis. We wondered whether IL‐1β or IL‐6 might influence the effect of TNF‐α as shown above. In the presence of IL‐1β or IL‐6, high numbers of TRAcP^+^ cells were present in monocyte cultures (Figure 3a). IL‐6 alone or a combination of IL‐6 and IL‐1β resulted in a significantly higher number of osteoclasts compared to the control cultures (with M‐CSF and RANKL alone) (Figure 3b). Monocytes proved to be insensitive to IL‐1β: no increased osteoclastogenesis was observed here; a finding in line with previous findings (Cao et al., 2016). However, whenever TNF‐α was added at the onset of the monocyte cultures, osteoclastogenesis was completely inhibited (see conditions 2–6 in Figure 3). This was apparent both in the absence and in the presence of IL‐1β and/or IL‐6, indicating that the latter two cytokines were not able to prevent the inhibitory effect of TNF‐α. In the presence of TNF‐α most cells proved to be TRAcP‐negative (Figure 3a, conditions 2–6). This inhibitory effect was not found with early blasts and myeloid blasts (Figure S2). Most of these cells became TRAcP^+^ multinucleated cells under all conditions (Figure S2a,c). With these two cell lineages, no inhibitory effect was found in any of the conditions (Figure S2b,d).

### 3.3 The inhibitory effect of TNF‐α did not occur when monocytes were seeded on bone

**Figure 4:**
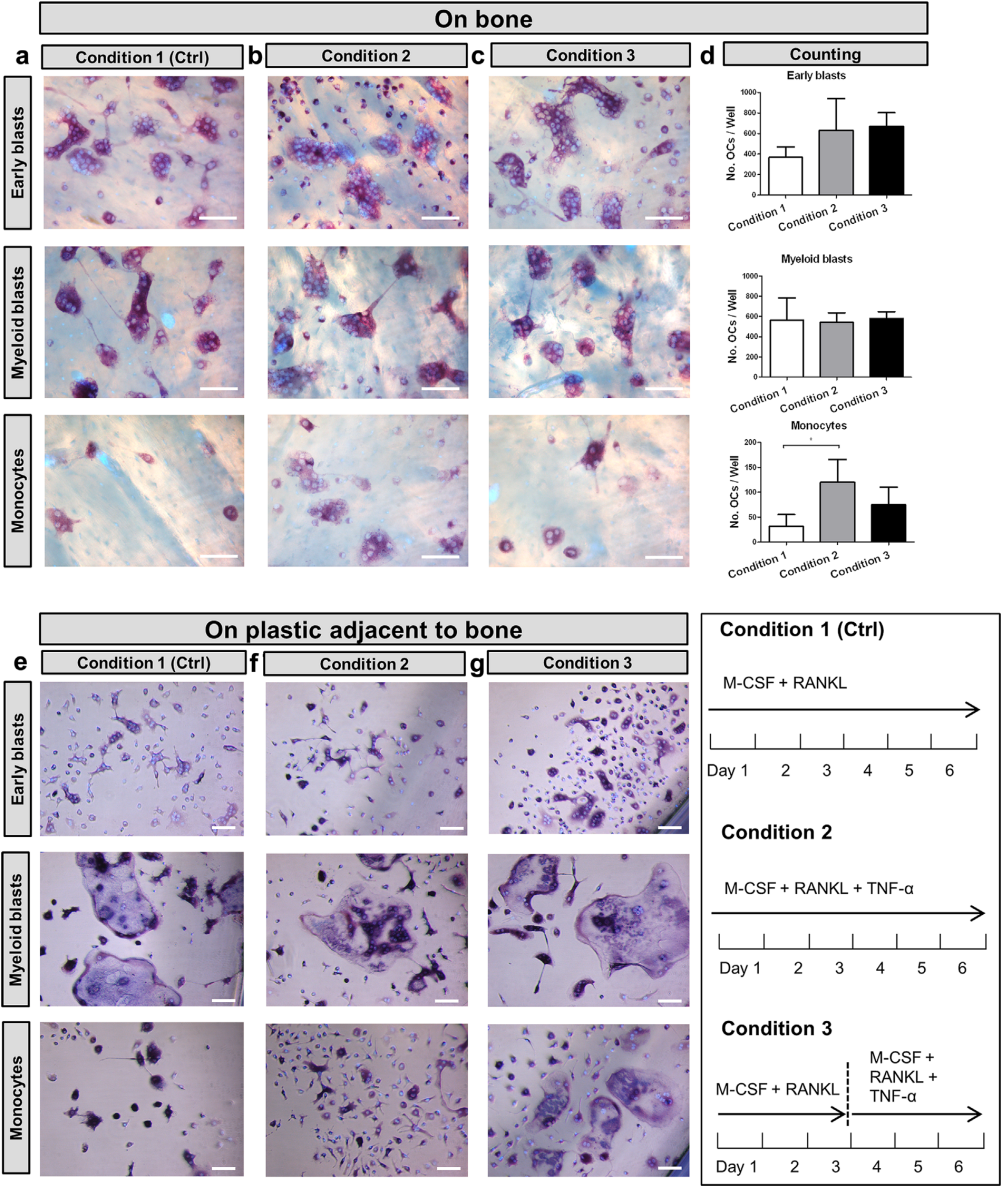
The inhibitory effect of TNF‐α on osteoclastogenesis was abolished by seeding monocytes on bone. Micrographs of cultures on bone of the three subsets. (a) condition 1 (control) with 30 ng/ml M‐CSF and 20 ng/ml RANKL for 6 days; (b) condition 2, with 30 ng/ml M‐CSF and 20 ng/ml RANKL with 10 ng/ml TNF‐α for 6 days; and (c) condition 3, with 30 ng/ml M‐CSF and 20 ng/ml RANKL first cultured for 3 days followed by 3 days culture with 30 ng/ml M‐CSF, 20 ng/ml RANKL, and 10 ng/ml TNF‐α. Scale bar = 100 μm. (d) Total number of TRAcP^+^ multinucleated cells (>2 nuclei). (*n* = 6, *p < 0.05). (e–g) Micrographs of the TRAcP^+^ cells formed on plastic next to bone slices of the same cultures. Cells were visualized by TRAcP staining (purple) and nuclei were counterstained by DAPI (blue). Note the presence of multinucleated TRAcP^+^ cells in monocyte cultures in condition 2. Scale bar = 100 μm

In addition to seeding the precursors on plastic, we also seeded them on bone slices (Figure 4), and cultured them (i) with M‐CSF and RANKL (Figure 4a); (ii) with TNF‐α, M‐CSF, and RANKL (Figure 4b); or (iii) primed them with M‐CSF and RANKL for 3 days followed by the addition of TNF‐α (Figure 4c). In contrast to the response on plastic, TNF‐α stimulated the formation of osteoclasts from monocytes when added at the start of the experiment on bone. This was only found with the monocytes, such a stimulation was not seen with early blasts and myeloid blasts on bone (Figure 4d). Also monocytes on the plastic adjacent to bone readily differentiated into TRAcP^+^ mononuclear and multinucleated cells (Figure 4f in monocyte panel). Cells on bone differentiated into a significantly higher number of osteoclasts than on plastic when cultured with M‐CSF, RANKL, and TNF‐α. This was found for all three precursor subsets (Figure S1g).

### 3.4 TNF‐α stimulated bone resorption

**Figure 5:**
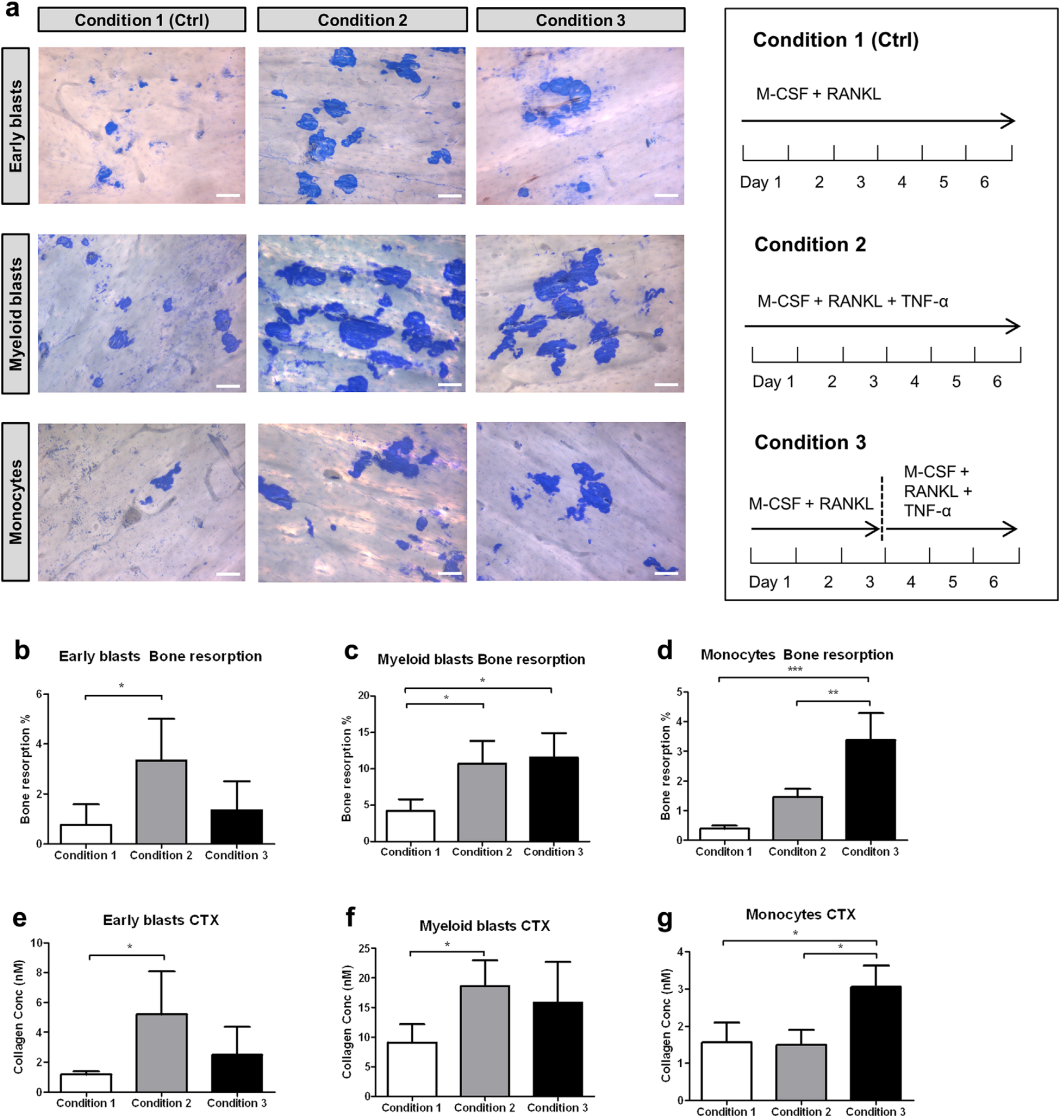
Bone resorption under the influence of TNF‐α. (a) Resorption visualized with Coomassie brilliant blue staining, in cultures with 30 ng/ml M‐CSF and 20 ng/ml RANKL (control) for 6 days (condition 1); 30 ng/ml M‐CSF and 20 ng/ml RANKL with 10 ng/ml TNF‐α for 6 days (condition 2), and with 30 ng/ml M‐CSF and 20 ng/ml RANKL first cultured for 3 days followed by 3 days culture with 30 ng/ml M‐CSF, 20 ng/ml RANKL, and 10 ng/ml TNF‐α (condition 3). (b–d) Percentage of bone resorbed by osteoclasts derived from early blasts, myeloid blasts, and monocytes. (e–f) Collagen fragment release from early blast cultures, myeloid blast cultures, and monocyte cultures measured with CTX assay after 6 days. Scale bar = 100 μm. (*n* = 6, *p < 0.05, **p < 0.01, ***p < 0.001)

To determine the level of bone resorption, the bone slices were stained with Coomassie brilliant blue to visualize pit formation, and the culture media were analyzed for the level of CTX (Figure 5). All bone slices showed resorption pits, indicating that under all conditions active osteoclasts were generated (Figure 5a). The level of resorbed bone was assessed by analyzing the surface area of the pits in relation to the entire bone surface and was expressed as percentage of resorption (Figure 5b–d). The data indicated that TNF‐α stimulated resorption by osteoclasts formed by early blasts (Figure 5b). Osteoclasts derived from monocytes showed the highest resorption when TNF‐α was added after M‐CSF and RANKL priming (Figure 5d). Osteoclasts generated from myeloid blasts, showed a significantly higher resorption area in both conditions with TNF‐α (Figure 5c). The CTX data were in line with the resorption data and confirmed the stimulatory effect of TNF‐α on bone resorption (Figure 5e–g).

### 3.5 TNF‐α down‐regulated osteoclast‐related genes and up‐regulated TNF‐α receptors by monocytes on plastic

**Figure 6:**
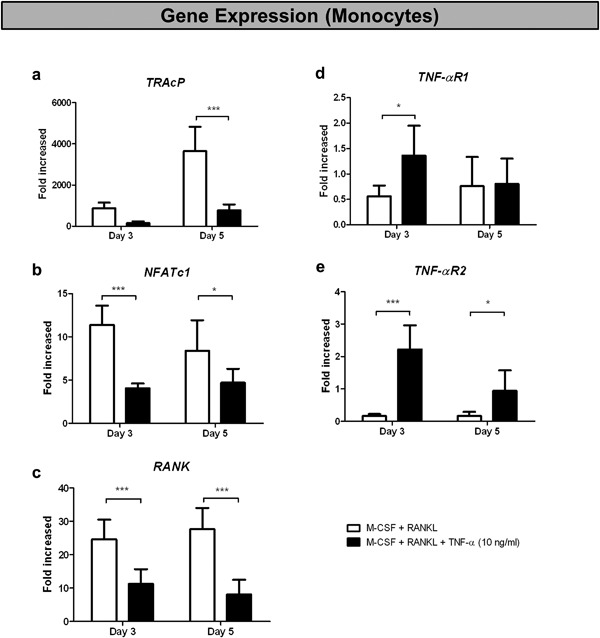
TNF‐α down‐regulated osteoclast‐related genes and up‐regulated TNF‐α receptors in monocyte cultures on plastic. Fold increase (compared to day 0) of genes (a) TRAcP, (b) NFATc1, (c) RANK, (d) TNF‐α R1, and (e) TNF‐α R2, of monocyte cultures on plastic. Cells were cultured on plastic either with 30 ng/ml M‐CSF and 20 ng/ml RANKL (white column) or with 30 ng/ml M‐CSF, 20 ng/ml RANKL and 10 ng/ml TNF‐α (black column). The cultures were stopped at day 3 or day 5. (*n* = 6, *p < 0.05, **p < 0.01, ***p < 0.001)

In an attempt to offer an explanation for the inhibitory effect of TNF‐α on osteoclastogenesis of monocytes, we analyzed gene expression of osteoclast‐related genes, TRAcP, NFATc1, and RANK as well as the TNF‐α receptors, TNF‐ αR1, and TNF‐αR2. The expression of TRAcP (Figure 6a), NFATc1 (Figure 6b), and RANK (Figure 6c), were significantly down‐regulated in the presence of TNF‐α. The expression of TNF‐αR1 was significantly increased at day 3, whereas no difference was seen at day 5 (Figure 6d). The expression of the other receptor of TNF‐α, TNF‐αR2, was strongly up‐regulated both at day 3 and day 5 under the influence of TNF‐α (Figure 6e).

The expression of TRAcP, NFATc1, and RANK by myeloid blasts and early blasts was not affected by TNF‐α (Figure S3a–c, f–h). No significant differences were seen for any of the tested genes in early blast cultures (Figure S3a–e). In myeloid blasts an up‐regulation of TNF‐αR2 was seen (Figure S3j). At the onset of the culture period monocytes showed a significantly higher expression of TNF‐αR1 as well as TNF‐αR2 than the other two subsets (Figure S3l–m). All three subsets expressed comparable RANK at the start of the culture period (Figure S3k).

### 3.6 TNF‐α diminished the uptake of fluorescently labeled RANKL in monocyte cultures

**Figure 7:**
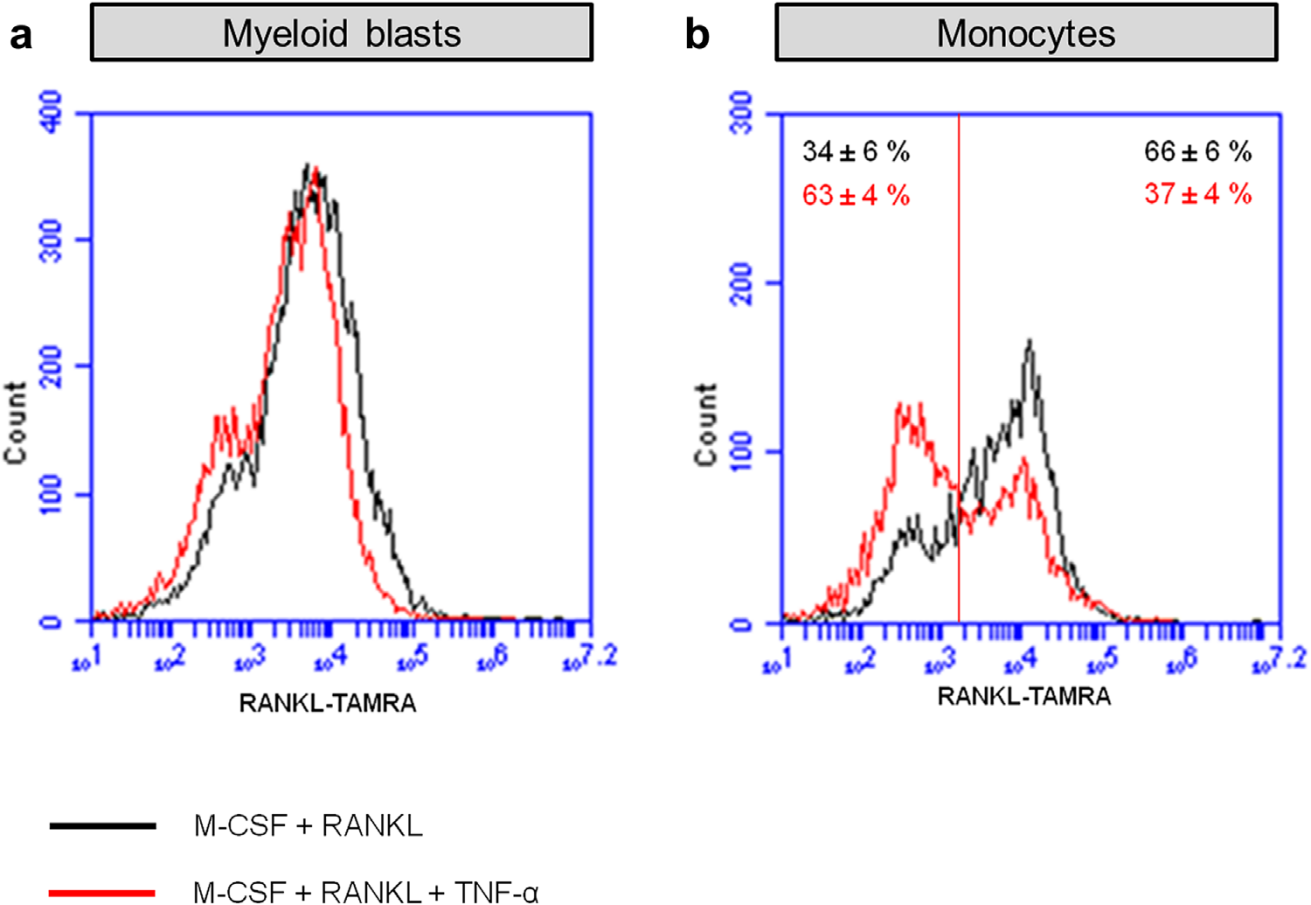
FACS analysis showed a different distribution of RANKL expression induced by TNF‐α in monocytes. (a) Binding of TAMRA‐labeled‐RANKL to myeloid blasts. (b) Binding of TAMRA‐labeled‐RANKL to monocytes. Black line: cells were cultured for 72 hr in the presence of 30 ng/ml M‐CSF and 20 ng/ml RANKL (control); red line: cells were cultured for 72 hr in the presence of 30 ng/ml M‐CSF, 20 ng/ml RANKL, and 10 ng/ml TNF‐α

In order to assess whether TNF‐α could interfere with binding of RANKL to RANK, FACS analysis was used to investigate the interaction of RANKL with the membrane‐bound receptor RANK. Monocytes and myeloid blasts were cultured for 3 days with M‐CSF and RANKL, with or without TNF‐α before labeling. The expression of RANK did not show a significant difference with or without TNF‐α in any of the cultures (data not shown). By adding TAMRA‐conjugated RANKL, we were able to analyze the binding of RANKL to its receptor RANK on the plasma membrane. TAMRA‐conjugated RANKL labeling of myeloid blasts was not influenced by the presence of TNF‐α (Figure 7A). In monocyte cultures, we found two populations with a different binding of labeled RANKL: one with a low binding (RANKL^low^) showed on the left hand side of Figure 7b, and one with a high binding (RANKL^high^), seen on the right hand side of the graph (Figure 7b). In the control group, the RANKL^high^ sub‐population was almost two times higher (66 ± 6%) than the RANKL^low^ sub‐population (34 ± 6%). In the group with TNF‐α, this pattern was reversed. The percentage of RANKL^high^ sub‐population was much lower (37 ± 4%) than that of RANKL^low^ sub‐population (63 ± 4%). Thus, TNF‐α decreased the binding of fluorescently labeled RANKL; an effect particularly apparent in monocytes.

## 4 DISCUSSION

In the present study we found that TNF‐α had the capacity to prevent osteoclastogenesis of one specific precursor subset: bone marrow derived monocytes (CD31^−^ Ly‐6C^hi^). Our results indicate that TNF‐α interferes with the binding of fluorescently labeled RANKL in monocytes. This likely results in the impairment of the RANK‐induced signaling pathway for osteoclastogenesis. However, this inhibitory effect can be prevented in different ways. First, by the presence of M‐CSF and RANKL prior to the addition of TNF‐α. Second, by seeding monocytes on bone. These phenomena were not found with early blast cultures nor with myeloid blast cultures. Our findings indicate a multifunctional role of TNF‐α on osteoclastogenesis of a particular subset of osteoclast precursors, the monocytes.

### 4.1 A model for TNF‐α on monocytes’ osteoclastogenesis

**Figure 8:**
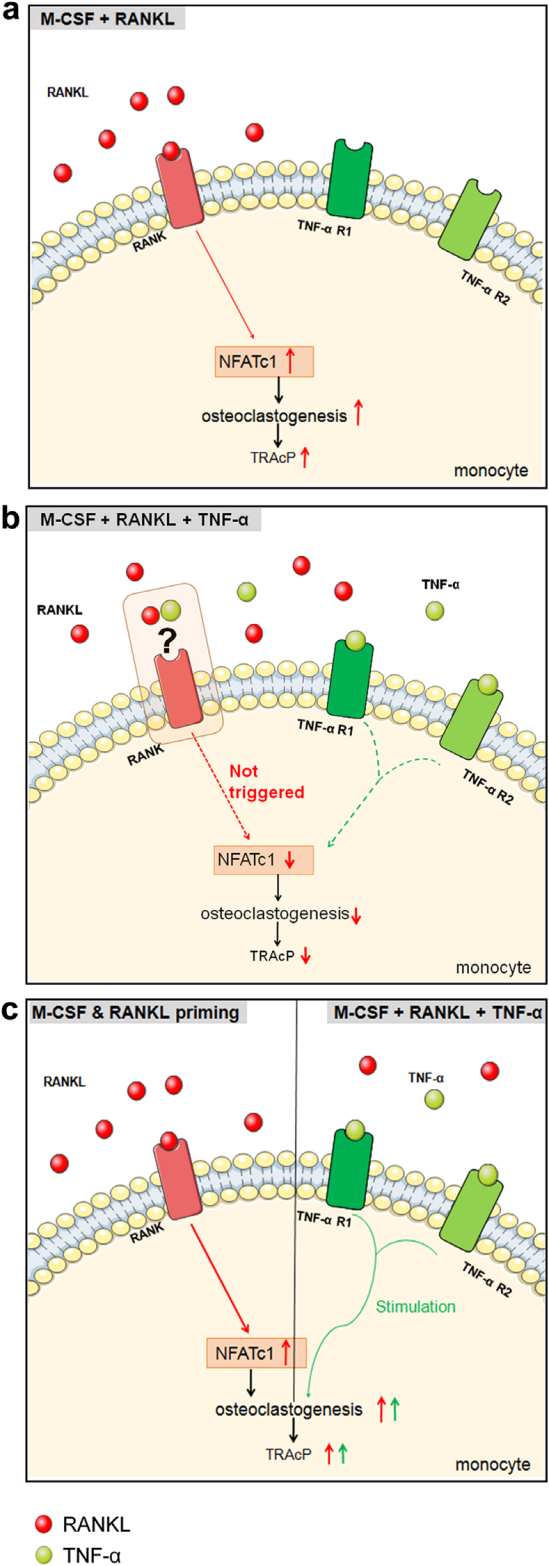
Hypothetical mechanism of the inhibitory effect of TNF‐α in monocyte cultures. (a) When monocytes are cultured in the presence of M‐CSF and RANKL (control), RANKL binds to RANK expressed on the membrane, up‐regulates NFATc1, and results in the stimulation of the osteoclastogenesis signaling pathway, leading to osteoclast differentiation. (b) When monocytes are cultured with M‐CSF, RANKL and TNF‐α, RANKL/RANK signaling is not triggered properly, leading to a down‐regulation of NFAcT1 and TRAcP and an inhibition of osteoclast formation. (c) When monocytes are incubated first with M‐CSF and RANKL for 3 days, RANK signaling is triggered. The addition of TNF‐α stimulates osteoclastogenesis

The most intriguing finding of this study was the inhibitory effect of TNF‐α on osteoclastogenesis of monocytes when cultured on plastic, whereas it increased osteoclastogenesis when the cells were first exposed to M‐CSF and RANKL or, alternatively, seeded on bone. Under the former (on plastic) condition we found a decreased mRNA level of RANK by the monocytes, and an increased population of monocytes with a lower level of fluorescently labeled RANKL. The protein level of RANK proved to be unaffected. Thus, it is likely that the RANKL/RANK signaling pathway was affected. Our findings indicate that TNF‐α can express both inhibitory and stimulatory effects on monocytes’ osteoclastogenesis. A hypothetical model that offers an explanation for our findings is shown in Figure 8.

When monocytes are cultured with M‐CSF and RANKL, RANKL will bind to RANK expressed on the membrane of monocytes, leading to up‐regulation of osteoclast‐related genes, such as NFATc1. This will result in osteoclast differentiation (Figure 8a). However, when TNF‐α is present from the onset together with M‐CSF and RANKL, it appears that RANK signaling is not well triggered (Figure 8b). This results in a down‐regulated gene expression of RANK, NFATc1, and TRAcP (Figure 6a–c), and as a consequence TRAcP‐negative mononuclear cells are formed (Figure 2b in monocyte panel). How TNF‐α interferes with RANK signaling remains to be elucidated. This block of osteoclast formation is not present when monocytes are first cultured with M‐CSF and RANKL followed by an addition of TNF‐α (Figure 8c). Under this condition, TNF‐α enhances the osteoclast formation by monocytes. We propose that TNF‐α if present at a later stage of osteoclast differentiation, can stimulate RANKL‐induced osteoclast generation.

### 4.2 Relationship between TNF‐α and RANK/RANKL

Clearly the relationship between TNF‐α and RANKL in osteoclastogenesis is complex. Kobayashi et al. (2000) showed that the stimulatory effect of TNF‐α on osteoclastogenesis is M‐CSF‐dependent rather than RANK/RANKL‐dependent. Studies by Kim et al. (2005) demonstrated that TNF‐α together with TGF‐β stimulates osteoclastogenesis independent of the RANKL‐RANK axis. However, Zhang et al. (2001) showed that TNF‐α synergistically cooperates with RANK/RANKL‐induced osteoclastogenesis and an overlapping signaling pathway of RANKL and TNF‐α was proposed. Others stated that RANKL‐ and TNF‐induced osteoclastogenesis share a similar intracellular signaling pathway, including c‐fos and NFATc1 (Yamashita et al., 2007). Lam et al. (2000) showed that a basal level of RANKL is necessary for TNF‐α induced osteoclast formation: TNF‐α alone, or with M‐CSF, at any concentration, failed to stimulate osteoclast differentiation. Depending on the time point, TNF‐α proved to inhibit or potentiate RANKL‐mediated osteoclastogenesis. TNF‐α inhibited osteoclastogenesis by purified myeloid cell cultures when it was present with RANKL at the onset of the culture, but the cytokine stimulated osteoclastogenesis when it was added 2–4 days after RANKL priming (Lam et al., 2000). This coincides with the present study which shows adding TNF‐α at a later time point after RANKL and M‐CSF priming stimulates monocyte‐derived osteoclast formation.

In order to prove that TNF‐α is closely associated with RANK/RANKL interaction, we cultured the three osteoclast precursor subsets with only M‐CSF for 3 days before adding TNF‐α together with M‐CSF and RANKL. Under these conditions only very few TRAcP^+^ multinucleated cells were formed (Figure S1d). This indicates that M‐CSF alone is not sufficient; a RANKL exposure is needed for the TNF‐α‐ induced osteoclastogenesis. To assess if TNF‐α can substitute RANKL in osteoclastogenesis, we also cultured the three osteoclast precursor subsets with M‐CSF and TNF‐α (Figure S1e,f). Interestingly, high number of TRAcP^+^ mononuclear cells were formed in early blast and myeloid blast cultures (Figure S1e), but only a few of them were multinucleated (Figure S1f). Almost no TRAcP^+^ cells were present in monocyte cultures (Figure S1e,f). This shows that TNF‐α can partly substitute RANKL in early blasts’ and myeloid blasts’ osteoclastogenesis, but not with monocytes.

### 4.3 Bone's stimulatory effect on osteoclastogenesis

The inhibitory effect of TNF‐α on osteoclastogenesis by monocytes was prevented not only with an M‐CSF and RANKL pre‐incubation, but also by seeding monocytes on bone slices. Under these conditions even higher numbers of osteoclasts were formed with TNF‐α. The data indicate that attachment of the monocytes to the bone surface changes the sensitivity of these cells to TNF‐α. Although we do not have an explanation for such an effect, several authors have shown that an interaction of osteoclast precursors with bone greatly affects release of compounds like IL‐1 (Yao, Xing, Qin, Schwarz, & Boyce, 2008). An increased level of IL‐1 has been shown to stimulate TNF‐ induced osteoclastogenesis (Wei, Kitaura, Zhou, Ross, & Teitelbaum, 2005). In our study, exposure to IL‐1β could not overcome the inhibitory effect found in monocytes on plastic (Figure 3). Therefore, key molecules of bone, or released by osteoclasts that were grown on bone, remain to be elucidated.

When we compare the number of osteoclasts per surface area when cells were cultured either on bone slices or on plastic plates, the number of osteoclasts formed on the bone slices were shown to be significantly higher than the number formed on plastic (Figure S1g). This is in line with our previous study in which we showed higher numbers of TRAcP^+^ multinucleated cells on bone than on plastic (Cao et al., 2016). Of considerable interest was the finding that cells present on plastic next to the bone slices did form osteoclasts. This counts also for monocyte cultures (Figure 4f in monocyte panel). Since monocytes seeded on plastic in the absence of bone were inhibited in their capacity to generate osteoclasts, this finding suggests the presence of one or more compounds prevented the TNF‐α induced inhibition. Such compounds could be released either by the monocytes due to their interaction with the bone surface or by the bone slice itself. Bone tissue, as a natural matrix for osteoclast formation, was shown to overcome osteoclastogenesis‐insensitivity of certain osteoclast precursor subsets (De Vries et al., 2015). It was demonstrated that expression of NFATc1, being a key molecule in the regulation of osteoclastogenesis, was stimulated on bone. Osteoclast precursors are likely to interact with a number of stimulatory components elicited from the bone matrix, for example transforming growth factor‐β (Mundy & Bonewald, 1990; Zwerina et al., 2004) and proteins such as osteopontin and bone sialoprotein (Fisher, Torchia, Fohr, Young, & Fedarko, 2001; Qin, Baba, & Butler, 2004, Yao et al., 2008). Molecules such as osteoclast‐associated receptor (OSCAR) expressed by pre‐osteoclasts, were shown as a collagen receptor that stimulates osteoclastogenesis (Barrow et al., 2011). All of these molecules have the potential to stimulate osteoclast generation. These findings suggest that the release of factors by osteoclast precursors or by the bone matrix may overcome the TNF‐α inhibitory effect. Further investigations are needed to explore the nature of the molecule(s) released.

### 4.4 Monocytes: a distinct osteoclast precursor cell lineage?

This study showed that from all the different bone marrow derived osteoclast precursors, only the monocyte‐derived osteoclast formation could be abolished by TNF‐α, a phenomenon that does not apply to early blast‐ or myeloid blast‐ derived osteoclast formation. A previous study already addressed the differences among the three subsets in osteoclast differentiation (De Vries et al., 2009) and showed myeloid blasts being the most responsive cells to M‐CSF and RANKL (De Vries et al., 2009). Both myeloid blasts and early blasts but not monocytes were shown to be sensitive to IL‐1β (Cao et al., 2016). In line with the inhibitory effect of TNF‐α which was only found in monocyte cultures in this study, Hayashi et al. (2003) showed that splenic osteoclastogenesis was completely inhibited by TNF‐α; an effect not found with bone marrow cells. Since the spleen stores about half of the number of monocytes present in the body (Swirski et al., 2009), our finding of the inhibitory effect of TNF‐α on bone marrow monocytes suggests a similarity between spleen monocytes and those present in the marrow. Comparing the three myeloid precursor subsets, monocytes showed a different distribution of the receptors of TNF‐α: monocytes have a significantly higher mRNA level of TNF‐αR1 and TNF‐αR2 on day 0 compared to myeloid blasts and early blasts (Figure S3l,m). This suggests that monocytes are the cell type more sensitive to TNF‐α, and this sensitivity has a close relationship to RANKL exposure.

**Figure 9:**
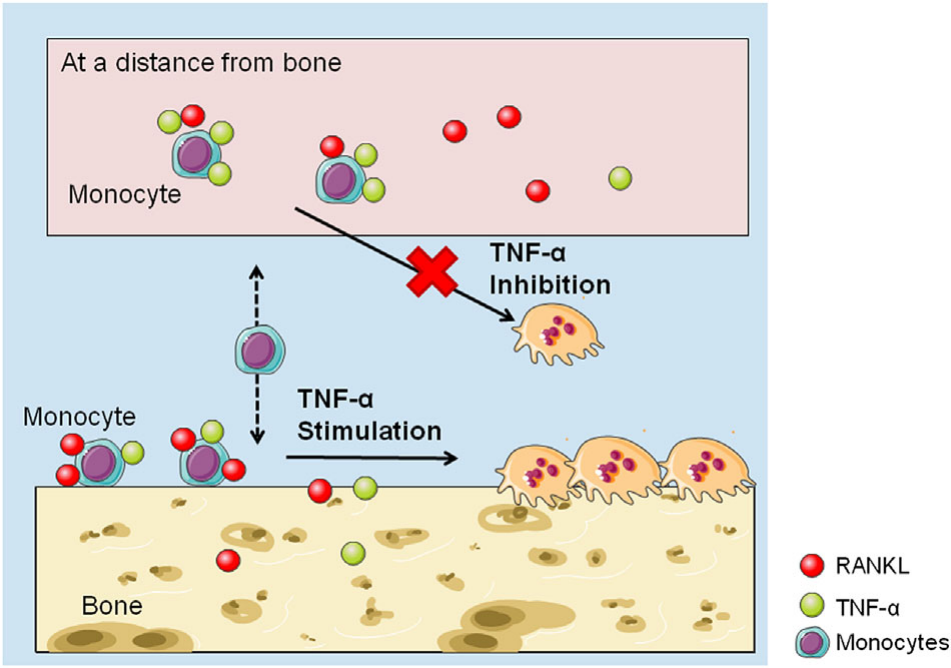
Physiological explanation of the effect of TNF‐α on monocytes’ osteoclastogenesis in vivo. TNF‐α inhibits monocytes’ osteoclastogenesis at sites at a distance from bone. When monocytes interact with the bone surface, TNF‐α stimulates RANKL‐induced osteoclast formation

Is there a physiological explanation for a cell type that becomes osteoclastogenesis‐insensitive to a cytokine like TNF‐α? Under pathological conditions the cytokine is present throughout the body and this could in theory result in the formation of osteoclasts anywhere in the body. After all TNF‐α as well as the monocytes are present at many different sites, and RANKL is also present in serum (Findlay & Atkins, 2011). We propose that monocytes, not in the vicinity of bone, are insensitive to the cytokine, therefore, osteoclast formation does not occur at these sites away from the bone (Figure 9). When these monocytes attach to the bone surface, they become sensitive to TNF‐α and osteoclastogenesis is stimulated. We hypothesize that such a versatile role of the cytokine is meaningful in modulating the process of osteoclastogenesis.

## Supporting information

Additional Supporting Information may be found online in the supporting information tab for this article.

Supporting Figures S1.
