# Supplementary material for: TNF‐α has both stimulatory and inhibitory effects on mouse monocyte‐derived osteoclastogenesis
Source: J Cell Physiol. 2017 Jul 17;232(12):3273–85. doi: 10.1002/jcp.26024 (PMC5601245; doi:10.1002/jcp.26024)
Supplement: Supplementary file 1 — Supporting Figures S1. [file JCP-232-3273-s001.pdf]

Figure S1

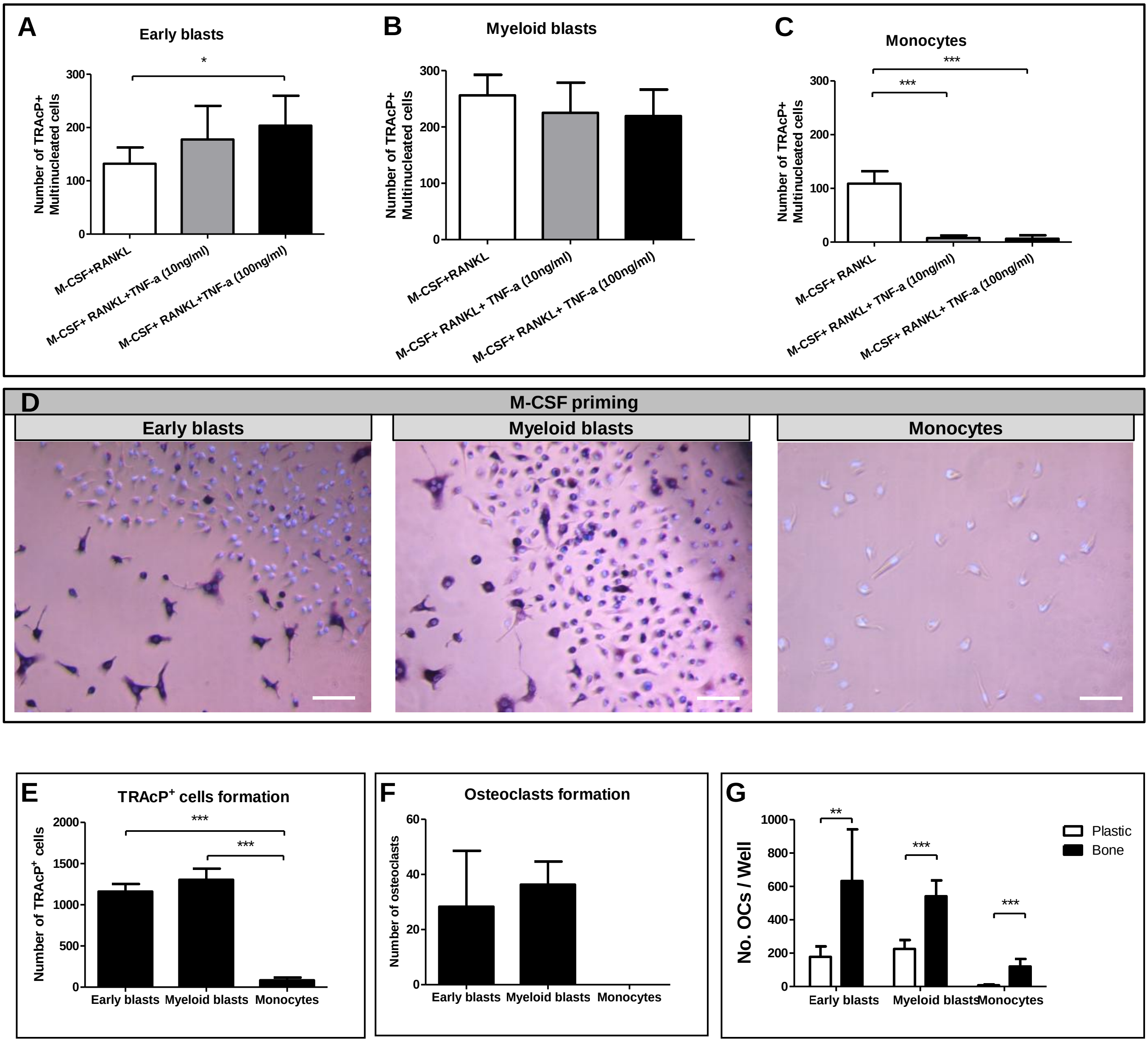

Figure S1. Osteoclast formation under different culture conditions. A-C. No significant differences were found in osteoclast formation between different concentrations of TNF-α. Early blasts (A), myeloid blasts (B) and monocytes (C) were cultured with 30 ng/ml M-CSF and 20 ng/ml RANKL (white column); or with 30 ng/ml M-CSF, 20 ng/ml RANKL and 10 ng/ml TNF- α (grey column); or with 30 ng/ml M-CSF, 20 ng/ml RANKL and 100 ng/ml TNF-α (black column) for 5 days on plastic. The total number of TRAcP$^+$ multinucleated cells (>2 nuclei) were counted as osteoclasts. (D) M-CSF priming did not trigger osteoclast precursors to respond to TNF-α. Micrographs are of the three subsets cultured with only 30 ng/ml M-CSF for 3 days before adding 10 ng/ml TNF-α, 30 ng/ml M-CSF and 20 ng/ml RANKL for another 2 days. Scale bar= 100 μm. E-F. TNF-α drove RANKL independent osteoclastogenesis in early blast and myeloid blast cultures, but not in monocyte cultures. Early blasts, myeloid blasts and monocytes were cultured with only 30 ng/ml M-CSF and 10 ng/ml TNF-α on plastic for 5 days. Total number of TRAcP$^+$ cells (including the mononuclear cells) (E) as well as the number of osteoclasts (nuclei >2 TRAcP$^+$ cells) (F) were counted. There were almost no TRAcP$^+$ cells visible in monocyte cultures. G. Cells cultured on bone slices gave significantly higher number of osteoclasts than cultured on plastic. Cells were cultured with 30 ng/ml M-CSF, 20 ng/ml RANKL and 10 ng/ml TNF-α on plastic for 5 days (white column) or on bone slices for 6 days (black column) (n=6, * P<0.05, ** P<0.01, ***P<0.001).

Figure S2

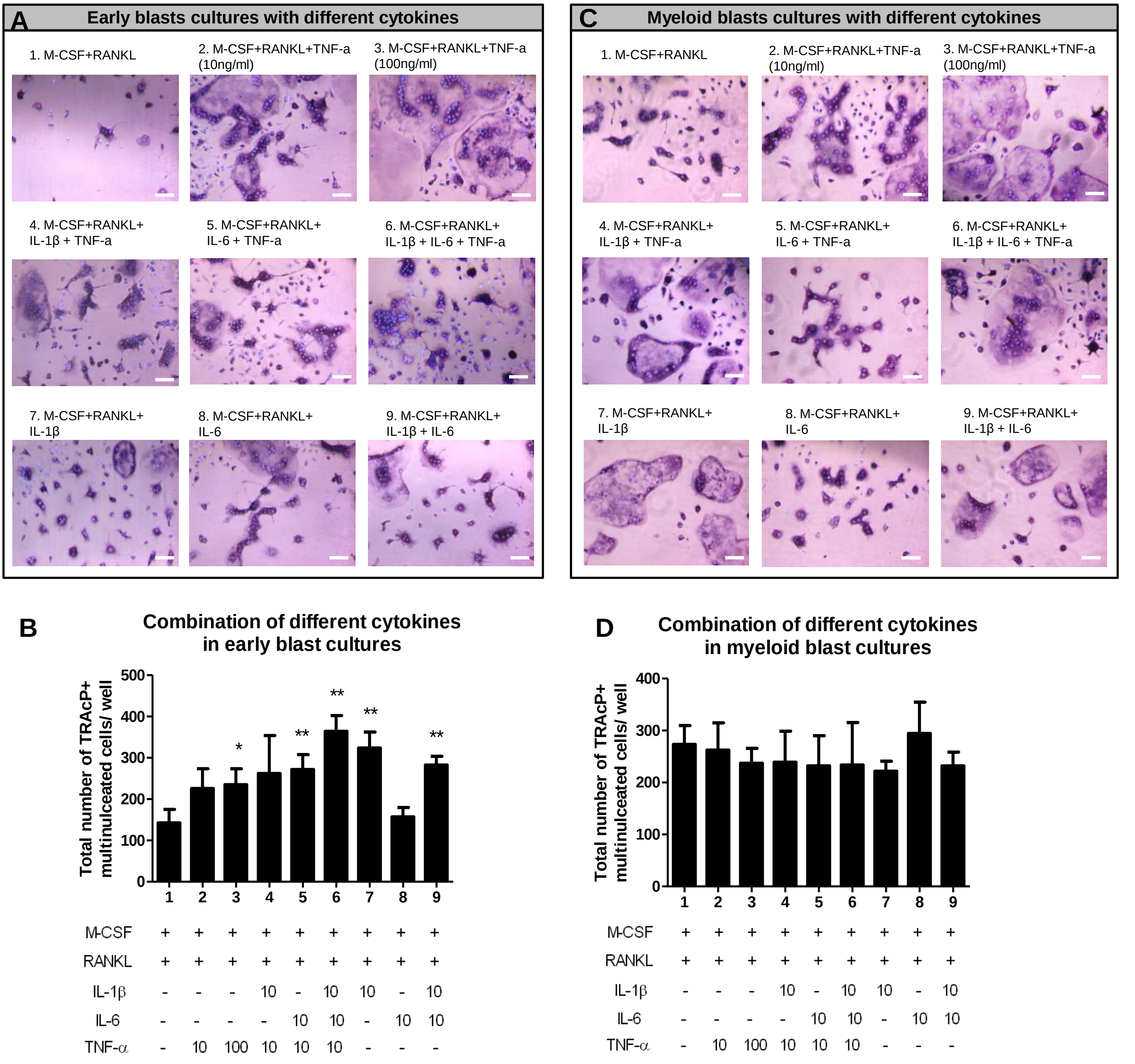

Figure S2. Effects of different cytokines on early blasts and myeloid blasts. (A) Micrographs of early blast cultures on plastic with the combination of different cytokines. (B) Total number of TRAcP+ multinucleated cells of these 9 different culture conditions in early blast cultures. (C) Micrographs of myeloid blast cultures on plastic with the combination of different cytokines. (D) Total number of TRAcP+ multinucleated cells of these 9 different culture conditions in myeloid blast cultures. Cells with >2 nuclei were counted as osteoclasts. Scale bar= 100 μm (n=6, * P<0.05, ** P<0.01).

Figure S3

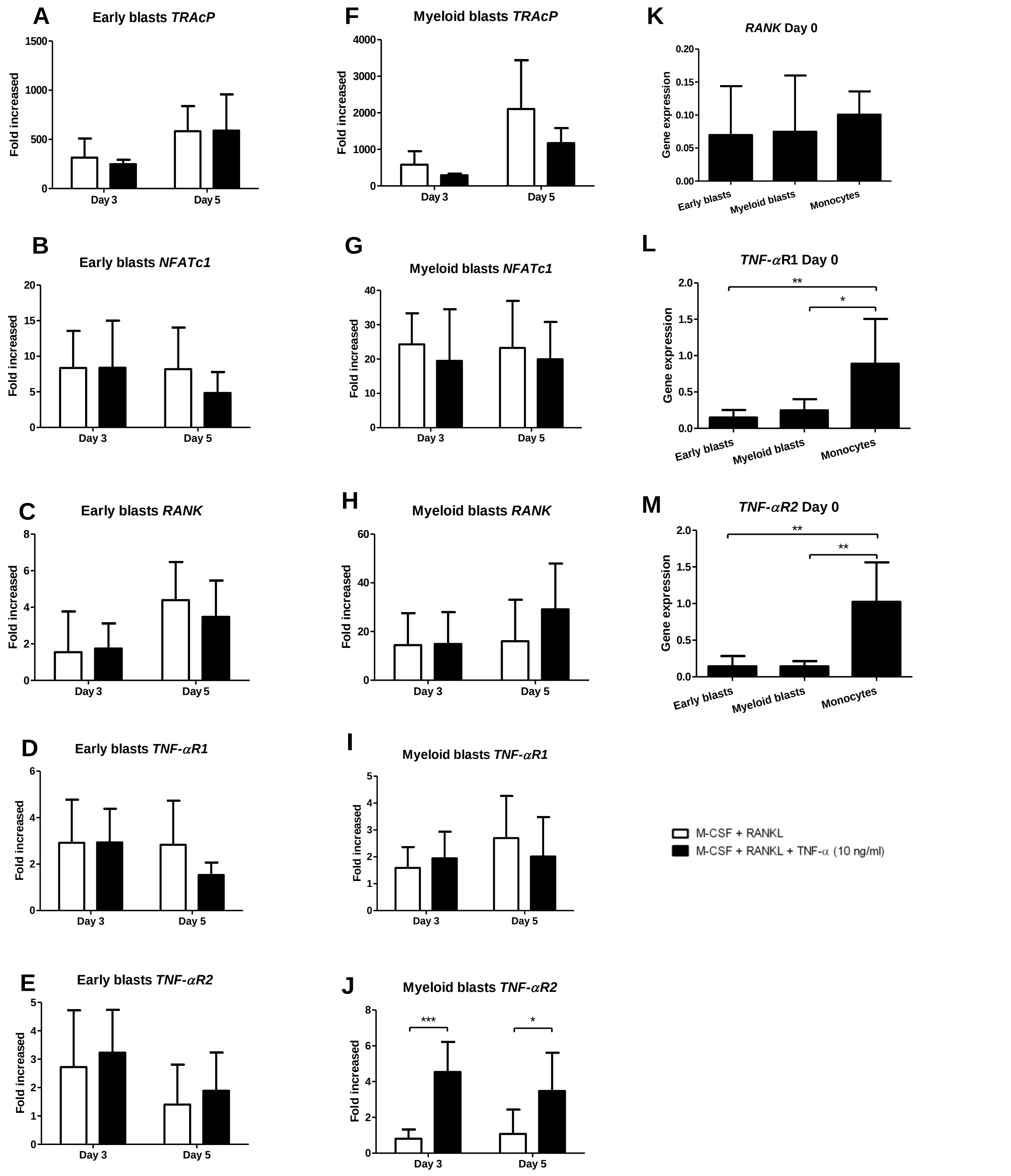

Figure S3. Gene expression. A-E. Fold increase (compared to day 0) of genes *TRAcP* (A), *NFATc1* (B), *RANK*(C), *TNF-αR1* (D) and *TNF-α R2* (E), of early blast cultures on plastic. F-J. Fold increase (compared to day 0) of the same genes, *TRAcP* (F), *NFATc1* (G), *RANK* (H), *TNF-αR1* (I) and *TNF-αR2* (J) expressed by myeloid blast cultures on plastic. Cells were either cultured on plastic with 30 ng/ml M-CSF and 20 ng/ml RANKL (white column) or cultured with 30 ng/ml M-CSF, 20 ng/ml RANKL and 10 ng/ml TNF-α (black column) and stopped at day 3 or day 5. K-M. Expression of *RANK* (K), *TNF-αR1* (L) and *TNF-αR2* (M) by early blasts, myeloid blasts and monocytes immediately after isolation (n=6 , * $P<0.05$, ** $P<0.01$, ***$P<0.001$).
